# Comparative mortality of hemodialysis patients at for-profit and not-for-profit dialysis facilities in the United States, 1998 to 2003: A retrospective analysis

**DOI:** 10.1186/1471-2369-9-6

**Published:** 2008-06-26

**Authors:** Robert N Foley, Qiao Fan, Jiannong Liu, David T Gilbertson, Eric D Weinhandl, Shu-Cheng Chen, Allan J Collins

**Affiliations:** 1 United States Renal Data System, 914 South 8th Street, Suite S-406, Minneapolis, Minnesota, USA; 2 Department of Medicine, Phillips-Wangensteen Building, University of Minnesota, Minneapolis, Minnesota, USA

## Abstract

**Background:**

Concern lingers that dialysis therapy at for-profit (versus not-for-profit) hemodialysis facilities in the United States may be associated with higher mortality, even though 4 of every 5 contemporary dialysis patients receive therapy in such a setting.

**Methods:**

Our primary objective was to compare the mortality hazards of patients initiating hemodialysis at for-profit and not-for-profit centers in the United States between 1998 and 2003. For-profit status of dialysis facilities was determined after subjects received 6 months of dialysis therapy, and mean follow-up was 1.7 years.

**Results:**

Of the study population (*N *= 205,076), 79.9% were dialyzed in for-profit facilities after 6 months of dialysis therapy. Dialysis at for-profit facilities was associated with higher urea reduction ratios, hemoglobin levels (including levels above 12 and 13 g/dL [120 and 130 g/L]), epoetin doses, and use of intravenous iron, and less use of blood transfusions and lower proportions of patients on the transplant waiting-list (*P *< 0.05). Patients dialyzed at for-profit and at not-for-profit facilities had similar mortality risks (adjusted hazards ratio 1.02, 95% CI 0.99–1.06, *P *= 0.143).

**Conclusion:**

While hemodialysis treatment at for-profit and not-for-profit dialysis facilities is associated with different patterns of clinical benchmark achievement, mortality rates are similar.

## Background

The incidence rate of treated end-stage renal disease (ESRD) has increased fourfold in the last quarter century [1]. In 2003, the cost to the US Medicare program for a typical dialysis patient was estimated at $67,000 and ESRD accounted for 6.7% of all Medicare expenditures, compared with 4.8% in 1991 [1]. Reimbursement for dialysis services, which has changed little since 1982, is delivered on a per-treatment basis, irrespective of medical, logistical, and infrastructure complexities; cost containment has been a concern since the early days of the Medicare ESRD program [2]. Not surprisingly, for-profit dialysis facilities have become the norm, with freestanding, private, chain-affiliated facilities exhibiting the most prolific growth [1].

The concern that treatment at for-profit dialysis facilities may be associated with lower survival rates has been debated for decades [3-12]. Two comparatively recent studies [8,12] demonstrated higher mortality rates at for-profit than at not-for-profit dialysis facilities, leading to national and international debate [13-17]. The first of these studies [8] examined a nationally representative sample of United States patients on hemodialysis at the end of 1990 and 1993; the second study [12] included patients from Michigan in 1973 through 1981, and patients on dialysis in the United States in 1990 and in 1993 through 1997. More recently, mortality was related to for-profit status in national random samples of patients receiving hemodialysis therapy in the United States at the beginning of 1994 through 2000 [18]. In the last of these studies, while unadjusted analysis showed no differences in mortality, adjustment for age, demography, cause of renal disease, and on-therapy clinical benchmarks showed higher mortality hazards ratio for patients treated at for-profit facilities [18]. The possibility that dialysis at for-profit facilities, where 4 of every 5 dialysis patients receive care [1] may be associated with a survival disadvantage has not been examined in more recent cohorts beginning dialysis therapy in the US. Our study was an attempt to address this issue.

## Methods

### Objectives

Our primary objective was to compare the mortality rates of patients starting hemodialysis at for-profit and at not-for-profit hemodialysis facilities in the United States between 1998 and 2003. Secondary objectives included comparison of clinical benchmarks according to for-profit or not-for-profit status.

### Design

The United States Renal Data System (USRDS) generally recommends beginning outcome analyses after 90 days have elapsed since the first dialysis treatment (the 90-day rule), partly to allow time to establish a stable dialysis choice and partly because in-center hemodialysis patients aged less than 65 years cannot bill Medicare for their dialysis treatments until 90 days have elapsed [1]. Thus, for this study, the starting date was the 91st day after dialysis inception. Two phases were then constructed, with the first 3 months of the study (the exposure period) used to characterize the study population, including assessment of clinical benchmarks, and subsequent follow-up time (the outcome period) used to assess mortality.

### Study Population

We used the 100% ESRD sample from the Medicare database to select patients who were first dialyzed between January 1, 1998, and December 31, 2003; had Medicare as primary payer throughout the exposure period; and were on hemodialysis at either a for-profit or not-for-profit dialysis facility at the end of the exposure period.

Patient demographics were obtained from the ESRD Medical Evidence Report (Centers for Medicare & Medicaid Services [CMS] form CMS-2728-U4), which is filed for all patients initiating maintenance dialysis. Medicare claims generated during the exposure period were used as supplementary data sources to identify comorbid conditions. Comorbid conditions from Medicare Part A institutional and Part B physician/supplier claims were identified by *International Classification of Diseases, Ninth Revision, Clinical Modification *(ICD-9-CM) codes and *Current Procedural Terminology *(CPT) codes. Comorbid conditions were considered present if an affirmative response was present in the Medical Evidence Report or on Medicare Part A or Part B claims. Cumulative hospital days and infectious hospitalization admissions in the exposure period were also determined from Medicare inpatient claim files. Clinical benchmarks, including hemoglobin levels, epoetin doses, urea reduction ratios, intravenous iron use, and blood transfusions, were obtained from Medicare institutional outpatient claims; wait-listing for renal transplant was obtained from the USRDS database. Vital status was obtained from the CMS ESRD Death Notification (form CMS-2746-U3), and renal transplantation from the USRDS database.

Dialysis facility profit status (for-profit or not-for-profit) and facility status (freestanding or hospital-based) were determined from the CMS Annual Facility Survey. Dialysis facilities can change their profit status, and patients can change dialysis facilities. We applied the USRDS 60-day collapsing rule to such changes, namely, that they must remain in place for at least 60 days to be considered stable [1].

### Analysis

Follow-up began immediately after the exposure period, and ended at the earliest occurrence of 3 years elapsed, death, renal transplantation, loss to follow-up, or December 31, 2004. The characteristics of patients receiving dialysis at for-profit and not-for-profit dialysis units were compared using the chi squared test for categorical variables and multivariate logistic regression. Cox proportional hazards regression was used to quantify the mortality hazards ratios. The robust standard error method was used to account for the possibility of clustering of patients within dialysis facilities [19]. All analyses were performed using SAS version 9.1 (SAS Institute, Inc., Cary, NC).

## Results

In all, 258,774 patients were eligible for analysis. Of these, facility characteristics were unknown for 26,455. For another 27,243 patients, information was lacking on date of birth, sex, race, primary renal disease, body mass index, or employment status. Hence, the final sample size was 205,076.

After 6 months of dialysis therapy, 79.9% of the sample was dialyzed at one of 3632 for-profit facilities, and 20.1% at one of 1264 not-for-profit facilities. Table 1 shows the characteristics of the overall study population and a comparison of patients in for-profit and not-for-profit facilities. On multivariate analysis, the characteristics associated with therapy at for-profit facilities were as follows: freestanding units; more recent calendar year; age ≤ 65 years; female sex; non-white race; overweight; fewer retirees; diabetes and hypertension as primary causes of renal disease; hospitalization days during the exposure period; fewer infectious hospitalizations; absence of atherosclerotic heart disease, dysrhythmia, chronic obstructive primary disorder, and cancer; and presence of congestive heart failure and hepatic disease.

**Table 1:** Baseline Characteristics

		Facility Profit Status				
					
Characteristic	All, 100.0% *N *= 205,076	Not-For-Profit, 20.1% *n *= 41,307	For-Profit, 79.9% *n *= 163,769	P*	AOR For-Profit (95% CI)	*P*^†^
Facility affiliation				< 0.0001		
Freestanding	12.9	41.4	98.6		Reference	-
Hospital-based	87.1	58.6	1.4		0.01 (0.01–0.01)	< 0.0001
Year of dialysis inception				< 0.0001		
1998	14.7	18.5	13.7		Reference	-
1999	15.4	17.6	14.8		1.16 (1.10–1.23)	< 0.0001
2000	16.6	17.8	16.3		1.12 (1.06–1.19)	< 0.0001
2001	17.0	16.0	17.3		1.06 (1.00–1.12)	0.032
2002	17.8	15.6	18.3		0.87 (0.83–0.92)	< 0.0001
2003	18.4	14.4	19.5		0.89 (0.85–0.94)	< 0.0001
Age group (years)				< 0.0001		
≤ 40	7.1	7.7	7.0		Reference	-
40 to 65	33.7	32.1	34.1		1.10 (1.04–1.17)	0.002
> 65	59.1	60.2	58.9		1.14 (1.06–1.22)	0.0002
Sex				< 0.0001		
Male	52.0	53.3	51.7		0.98 (0.95–1.01)	0.280
Female	48.0	46.7	48.3		Reference	-
Race				< 0.0001		
White	62.6	64.9	62.0		Reference	-
Black	32.4	29.1	33.2		0.91 (0.88–0.95)	0.001
Other	5.1	5.9	4.8		0.96 (0.90–1.03)	0.297
Body mass index (kg/m^2^)				< 0.0001		
< 18.5	5.9	6.4	5.8		0.90 (0.84–0.96)	0.001
18.5 to < 25	38.7	39.0	38.6		Reference	-
25 to < 30	28.3	28.2	28.3		1.02 (0.98–1.06)	0.358
≥ 30	27.1	26.4	27.3		1.04 (1.00–1.08)	0.074
Employment status				< 0.0001		
Employed	10.4	10.6	10.3		Reference	-
Unemployed	43.7	41.1	44.4		0.96 (0.91–1.01)	0.093
Retired	45.9	48.4	45.3		0.89 (0.84–0.94)	< 0.0001
Cause of ESRD				< 0.0001		
Diabetes mellitus	48.7	47.6	49.0		Reference	-
Hypertension	30.8	28.4	31.4		1.09 (1.05–1.13)	< 0.0001
Glomerulonephritis	7.9	9.1	7.6		0.91 (0.86–0.97)	0.002
Other	12.6	14.9	12.0		0.92 (0.87–0.96)	0.0003
Hospitalization (days)				< 0.0001		
0	65.9	65.6	66.0		Reference	-
0 to 5	15.6	15.1	15.7		1.04 (0.99–1.09)	0.110
> 5	18.5	19.3	18.3		1.16 (1.10–1.22)	< 0.0001
Infectious hospitalization	10.6	11.1	10.5	0.0011	0.94 (0.89–1.00)	0.036
Atherosclerotic heart disease	40.1	42.8	39.4	< 0.0001	0.93 (0.90–0.97)	0.0001
Congestive heart failure	45.0	46.2	44.7	< 0.0001	1.04 (1.00–1.07)	0.043
Stroke or TIA	16.0	16.5	15.9	0.0043	0.98 (0.94–1.02)	0.247
Peripheral vascular disease	27.5	29.4	27.1	< 0.0001	0.99 (0.96–1.03)	0.718
Dysrhythmia	18.8	20.5	18.3	< 0.0001	0.91 (0.87–0.95)	< 0.0001
Other cardiac disease	13.1	13.5	13.0	0.0223	1.02 (0.98–1.07)	0.347
COPD	14.0	15.4	13.6	< 0.0001	0.88 (0.84–0.92)	< 0.0001
Gastrointestinal disease	4.3	4.7	4.1	< 0.0001	0.96 (0.89–1.04)	0.311
Hepatic disease	8.6	6.8	9.1	< 0.0001	1.51 (1.42–1.60)	< 0.0001
Cancer	8.8	9.6	8.6	< 0.0001	0.94 (0.89–0.99)	0.022
Clinical benchmarks						
Urea reduction ratio (%)				< 0.0001		
< 60	8.3	9.3	8.1		0.93 (0.87–0.99)	0.019
60 to < 65	11.7	12.0	11.7		Reference	-
65 to < 70	21.7	20.0	22.1		1.20 (1.14–1.27)	< 0.0001
70 to < 75	24.3	21.0	25.2		1.22 (1.16–1.29)	< 0.0001
≥ 75	20.8	17.2	21.7		1.17 (1.11–1.24)	0.0002
Unknown	13.1	20.4	11.3		-	-
Hemoglobin (g/dL)^‡^				< 0.0001		
< 10	7.6	8.9	7.3		0.89 (0.84–0.95)	0.0002
10 to < 11	14.6	15.8	14.3		0.96 (0.92–1.01)	0.080
11 to < 12	28.7	28.7	28.6		Reference	-
12 to < 13	24.9	20.3	26.1		1.23 (1.19–1.29)	< 0.0001
≥ 13	13.3	9.3	14.3		1.66 (1.57–1.75)	< 0.0001
Unknown	10.9	16.9	9.3		-	-
Epoetin dose quartiles				< 0.0001		
(units/month)						
< 35,766	22.3	23.3	22.0		Reference	-
35,766 to < 58,200	22.4	22.3	22.4		0.96 (0.92–1.00)	0.050
58,200 to < 91,250	22.3	20.0	22.9		1.06 (1.02–1.11)	0.009
≥ 91,250	22.3	17.7	23.4		1.16 (1.10–1.21)	< 0.0001
Unknown	10.8	16.7	9.3		-	-
Intravenous iron use	71.1	60.1	73.9	< 0.0001	1.23 (1.19–1.27)	< 0.0001
Blood transfusion	6.3	6.8	6.2	< 0.0001	0.93 (0.87–0.99)	0.025
On transplant waiting list	2.7	3.6	2.5	< 0.0001	0.75 (0.69–0.82)	< 0.0001

Values are percentage of *n *in column head. AOR, adjusted odds ratio; COPD, chronic obstructive pulmonary disease; ESRD, end-stage renal disease; TIA, transient ischemic attack.

*Compared using the χ^2 ^test.

^†^Using multiple logistic regression, with adjustment for facility affiliation, year of dialysis inception, age group, sex, race, body mass index, employment status, cause of ESRD, days of hospitalization, infectious hospitalization, and comorbid conditions.

^‡^To convert hemoglobin in g/dL to g/L, multiply by 10.

Regarding clinical benchmarks after 6 months of dialysis therapy, on bivariate and multivariate analyses, patients at for-profit facilities had higher urea reduction ratios, higher hemoglobin levels (including levels above 12 and 13 g/dL [120 and 130 g/L]), more frequent use of intravenous iron, less frequent use of blood transfusions, and a lower proportion on the transplant waiting list (Table 1).

The overall crude mortality rate was 25.6 per 100 patient-years at risk, over a 1.7-year average duration of follow-up. Unadjusted mortality risk was higher for patients dialyzed at for-profit facilities (hazards ratio 0.89 compared with not-for-profit facilities, 95% confidence interval [CI] 0.87–0.92, *P *< 0.0001). Table 2 shows adjusted mortality associations from proportional hazards regression models. Patients dialyzed at for-profit and at not-for-profit facilities had similar mortality risks (adjusted hazards ratio [AHR] 1.02, 95% CI 0.99–1.06, *P *= 0.143). In contrast, mortality risk was higher for patients dialyzed at hospital-based facilities (AHR 1.18, 95% CI 1.14–1.23, *P *< 0.0001), compared with freestanding facilities.

**Table 2:** Mortality Hazards Ratios

Characteristic	Adjusted* Hazards Ratios (95% CI)	*P*
Facility profit status		
Not-for-profit	Reference	-
For-profit	1.02 (0.99–1.06)	0.143
Facility affiliation		
Freestanding	Reference	-
Hospital-based	1.18 (1.14–1.23)	< 0.0001
Year of dialysis inception		
1998	Reference	-
1999	1.00 (0.97–1.02)	0.707
2000	1.04 (1.01–1.07)	0.003
2001	1.03 (1.00–1.06)	0.035
2002	1.03 (1.00–1.06)	0.046
2003	1.01 (0.98–1.04)	0.724
Age group (years)		
≤ 40	Reference	-
40 to 65	1.50 (1.44–1.57)	< 0.0001
> 65	2.34 (2.23–2.45)	< 0.0001
Sex		
Male	0.97 (0.96–0.99)	< 0.0001
Female	Reference	-
Race		
White	Reference	-
Black	0.78 (0.77–0.80)	< 0.0001
Other	0.76 (0.72–0.81)	< 0.0001
Body mass index (kg/m^2^)		
< 18.5	1.21 (1.17–1.25)	< 0.0001
18.5 to < 25	Reference	-
25 to < 30	0.84 (0.82–0.85)	< 0.0001
≥ 30	0.76 (0.75–0.78)	< 0.0001
Employment status		
Employed	Reference	-
Unemployed	1.20 (1.15–1.24)	< 0.0001
Retired	1.22 (1.18–1.27)	< 0.0001
Cause of ESRD		
Diabetes mellitus	Reference	-
Hypertension	0.89 (0.88–0.91)	< 0.0001
Glomerulonephritis	0.73 (0.71–0.76)	< 0.0001
Other	0.99 (0.97–1.02)	0.490
Hospitalization (days)		
0	Reference	-
0 to 5	1.13 (1.11–1.16)	< 0.0001
> 5	1.54 (1.50–1.57)	< 0.0001
Infectious hospitalization	1.13 (1.10–1.16)	< 0.0001
Atherosclerotic heart disease	1.06 (1.04–1.08)	< 0.0001
Congestive heart failure	1.33 (1.31–1.36)	< 0.0001
Stroke or TIA	1.19 (1.17–1.21)	< 0.0001
Peripheral vascular disease	1.16 (1.14–1.17)	< 0.0001
Dysrhythmia	1.25 (1.23–1.28)	< 0.0001
Other cardiac disease	1.07 (1.05–1.10)	< 0.0001
COPD	1.23 (1.21–1.26)	< 0.0001
Gastrointestinal disease	1.21 (1.17–1.25)	< 0.0001
Hepatic disease	1.07 (1.03–1.10)	< 0.0001
Cancer	1.44 (1.41–1.48)	< 0.0001

COPD, chronic obstructive pulmonary disease; ESRD, end-stage renal disease. TIA, transient ischemic attack

*Using proportional hazards regression with adjustment for for-profit status, facility affiliation, year of dialysis inception, age group, sex, race, body mass index, employment status, cause of ESRD, days of hospitalization, infectious hospitalization, and comorbid conditions.

## Discussion

Using an inception cohort design spanning the years 1998 to 2003, we found similar mortality risks in patients dialyzed at for-profit and at not-for-profit facilities. For-profit status was associated with each of the clinical benchmarks studied. Thus, patients at for-profit facilities had higher urea reduction ratios, higher hemoglobin levels (including levels above recommended targets), more frequent use of intravenous iron, less frequent use of blood transfusions, and a lower proportion on the transplant waiting list.

With an average cost per dialysis patient to Medicare of $67,000 per year in 2002 [1] dialysis is undoubtedly an expensive therapy. The question of whether profit motives could compromise care for dialysis patients seems reasonable. Examining this issue regularly also seems reasonable, given that the treatment of dialysis patients continues to change rapidly. Recent national studies found associations between for-profit facility status and patient mortality different from the associations seen in this study. The first of these studies examined the question in a nationally representative sample of patients on hemodialysis in the United States at the end of 1990 and at the end 1993 [8]. The subset of patients receiving renal replacement therapy for more than 90 days and less than 1 year was chosen, and facility profit status was treated as a time-dependent variable. Treatment at a for-profit dialysis facility was associated with higher mortality hazards, the point estimate being 20% (95% CI 25–42%) higher than that in not-for-profit facilities [8].

The second study, a meta-analysis spanning 1973 to 1997, concluded that relative mortality rates were 8% higher at private, for-profit than at private, not-for-profit dialysis facilities [12]. The 8 studies included (4 peer-reviewed publications, 3 dissertations, 1 letter to an editor) were heterogeneous with regard to patient selection, covariate adjustment, and the methods used to generate comparative risk estimates. Twelve studies were not incorporated in the risk estimate because they included patients on treatment at public facilities and because the original authors were unable to perform analyses that excluded these patients. Interestingly, the overwhelming majority of patients considered for inclusion in the meta-analysis came from a single, publicly available dataset, the USRDS dataset. A *de novo *analysis of all available patients might provide useful information, such as homogeneous inclusion criteria and analytical methods, and the ability to include, exclude, or adjust for potential confounders, such as dialysis at public or private facilities. One potential explanation that could harmonize our findings with those from older studies is the possibility that quality of care has improved more in for-profit facilities over time than in not-for-profit or hospital-based facilities.

The most recent study related profit status to mortality in national random samples of US patients receiving hemodialysis therapy at the beginning of the years 1994 through 2000. Unadjusted analysis showed no mortality differences, but when adjustment was made for demography, cause of renal disease, and, notably, clinical benchmarks, higher mortality hazards ratios were seen for therapy at for-profit facilities; as in our study, patients in for-profit facilities had higher urea reduction ratios and hemoglobin values than those in not-for-profit facilities [18].

It is highly implausible that the primary research question addressed here could ever be addressed with a randomized controlled trial. That being said, the current study unquestionably suffers from all limitations inherent to observational designs. Thus, while identification of high-risk populations is possible, accurate delineation of causal pathways is not. Despite its limitations, we believe that this study offers useful information. The sample size was large, and a national-level population was examined over several years. Consequently, one methodology was applied consistently, to all patients, in all years. The study included relatively contemporary patient cohorts. It used publicly available data, so others can explore the validity of the approaches used, now and in the future.

## Conclusion

Our findings suggest that, in contemporary hemodialysis patients in the United States, treatment at for-profit and at not-for-profit dialysis facilities is associated with similar mortality rates.

## Competing interests

The authors declare that they have no competing interests.

## Authors' contributions

Study conception and design: RNF, JL, DTG, S–CC, AJC. Acquisition of data: S–CC, AJC. Analysis and interpretation of data: RNF, QF, JL, DTG, EDW, S–CC, AJC. Drafting the manuscript: RNF. Revising the manuscript critically for important intellectual content: QF, JL, DTG, EDW, S–CC, AJC.

## Pre-publication history

The pre-publication history for this paper can be accessed here:


